# Poly Ethylene Glycol (PEG)‐Based Hydrogels for Drug Delivery in Cancer Therapy: A Comprehensive Review

**DOI:** 10.1002/adhm.202300105

**Published:** 2023-05-01

**Authors:** Zihan Wang, Qinzhou Ye, Sheng Yu, Behnam Akhavan

**Affiliations:** ^1^ College of Chemistry Nankai University Tianjin 300071 P. R. China; ^2^ Sichuan Agricultural University Sichuan 611130 P. R. China; ^3^ Chemical Synthesis and Pollution Control Key Laboratory of Sichuan Province China West Normal University Nanchong 637000 P. R. China; ^4^ School of Engineering University of Newcastle Callaghan NSW 2308 Australia; ^5^ Hunter Medical Research Institute (HMRI) New Lambton Heights NSW 2305 Australia; ^6^ School of Physics The University of Sydney Sydney NSW 2006 Australia; ^7^ School of Biomedical Engineering The University of Sydney Sydney NSW 2006 Australia; ^8^ Sydney Nano Institute The University of Sydney Sydney NSW 2006 Australia

**Keywords:** cancer, drug delivery system, hydrogels, PEG, stimuli‐responsiveness

## Abstract

Hydrogel‐based drug delivery systems (DDSs) can leverage therapeutically beneficial outcomes in cancer therapy. In this domain, polyethylene glycol (PEG) has become increasingly popular as a biomedical polymer and has found clinical use. Owing to their excellent biocompatibility, facile modifiability, and high drug encapsulation rate, PEG hydrogels have shown great promise as drug delivery platforms. Here, the progress in emerging novel designs of PEG‐hydrogels as DDSs for anti‐cancer therapy is reviewed and discussed, focusing on underpinning multiscale release mechanisms categorized under stimuli‐responsive and non‐responsive drug release. The responsive drug delivery approaches are discussed, and the underpinning release mechanisms are elucidated, covering the systems functioning based on either exogenous stimuli‐response, such as photo‐ and magnetic‐sensitive PEG hydrogels, or endogenous stimuli‐response, such as enzyme‐, pH‐, reduction‐, and temperature‐sensitive PEG hydrogels. Special attention is paid to the commercial potential of PEG‐based hydrogels in cancer therapy, highlighting the limitations that need to be addressed in future research for their clinical translation.

## Graphical abstract

This article systematically and critically reviews the progress in the fabrication, characterization, and application of Poly Ethylene Glycol (PEG)‐based hydrogels for drug delivery in cancer therapy. The article sheds light on the design criteria that are important in regulating the on‐demand release of anti‐cancer drugs from PEG hydrogels in response to various stimuli. Special emphasis is placed on the commercial viability of these hydrogels, while also highlighting the critical limitations that must be overcome in future research for successful clinical translation.

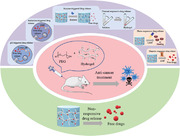

## 1 Introduction

Cancer is a catastrophic disease, annually causing the death of nearly 9 million people worldwide.^[^ ^1^, ^2^, ^3^ ^]^ The common approaches employed to treat cancer include surgery, chemotherapy, radiotherapy, and the recently developed immunotherapy.^[^ ^4^, ^5^, ^6^, ^7^ ^]^ These treatments have made promising progress in the control of cancer, with the life of many patients saved in recent years. In the US, for example, the rate of cancer‐related deaths has been steadily decreasing since it reached its highest point in 1991.^[^ ^8^ ^]^

Despite this promising progress in cancer therapy, the effective treatment of cancer is still a global grand challenge, and anti‐cancer drugs with high efficacy are urgently demanded.^[^ ^9^ ^]^ Indeed, chemotherapy that can be combined with immunotherapy has been the core treatment modality for cancer patients up to now. As such, a series of chemotherapeutic anti‐cancer drugs have emerged during the last two decades for experimental and clinical use.^[^ ^10^, ^11^, ^12^ ^]^ However, these drugs come with significant drawbacks, including short half‐life, wide bio‐distribution, non‐selectivity, low‐aggregated concentration in tumor tissue, and systemic toxicity.^[^ ^13^ ^]^

To improve the efficacy of chemotherapy and reduce the side effect of anti‐cancer drugs, a wide range of nanotechnology‐based drug delivery systems (DDSs) have been developed in recent years.^[^ ^14^, ^15^, ^16^ ^]^ An ideal DDS should be a multifaceted platform capable of comprehensively regulating the distribution of drugs in the biological body in terms of space, time, and dosage. The goal of DDSs lies in the delivery of the right amounts of drugs to the right place at the right time, thereby increasing the drug utilization efficiency and reducing toxicity.

Among an array of materials used in the drug delivery domain, hydrogels have shown great potential as highly promising biomaterials to develop multifaceted DDSs. Such hydrogel‐based materials have been extensively studied either in the lab or in the clinic. Notably, two hydrogel‐based products, Genexol®‐PM and OncoGel™ (**Table** **1**) have already been approved by Food and Drug Administration (FDA) and are currently used as clinical products.^[^ ^17^ ^]^

**Table 1:** Two main hydrogel‐based anti‐cancer nano drugs currently used in the clinic

Hydrogel‐based Clinical Product	Delivery System	Anti‐cancer Drug	Type of Cancer	Approved year	Company
Genexol®‐PM	Micelle polymer	Paclitaxel	Ovarian cancer and breast cancer	2009	Samyang
OncoGel™	Thermal‐sensitive ReGel® polymer	Paclitaxel	Esophageal Cancer	2007	Diatos

Hydrogels are made of a cross‐linked polymer network with a large content of water. Their degradability and eventual elimination from a biological system can be easily tuned by altering their degree of cross‐linking.^[^ ^16^ ^]^ Most hydrogel‐based materials have excellent biocompatibility, neglectable cytotoxicity, and prominent drug‐encapsulating capability,^[^ ^18^, ^19^ ^]^ making them appealing candidates as DDSs.

In regard to both active‐ and passive‐targeting drug delivery strategies, the hydrogel‐based DDSs offer imperative advantages. For active targeting drugs that are rapidly eliminated by the reticuloendothelial system and because of high interstitial fluid pressure in tumors, a hydrogel‐based system can extend the physical and chemical stability of chemotherapeutic agents. In general, a hydrogel‐based DDS can reduce the overall dose required and thus decrease the drugs’ side effects.^[^ ^20^, ^21^ ^]^

The ability of nanoparticles to accumulate in the tumor tissue highly depends on the vascular permeability of the tumor. Thus, the drugs’ accumulation at the tumor site via passive targeting (EPR effect) may not be as effective as the active targeting. Hydrogels that show stimuli‐sensitive properties can address this issue. By implantation of hydrogel in the tumor site, the loaded drugs can be locally delivered when needed in response to external stimuli such as temperature, pH, ionic strength, and light. This intriguing approach, enabled by the application of stimuli‐responsive hydrogels, enhances the efficacy of controlled‐release compared with a passive targeting strategy.^[^ ^22^, ^23^, ^24^ ^]^

Polyethylene glycol (PEG) has a long history of application as a building block of many biomaterials widely utilized in biomedical engineering fields, such as tissue regeneration and drug delivery. PEG‐based biomaterials come with an array of noteworthy virtues like biocompatibility, no immune response stimulation, and great solubility in water. These properties have made PEG a highly appealing biomaterial applied in a range of formats, including bulk, thin solid films, hydrogels, and nano particles, as summarized in **Table** **2**.

**Table 2:** Biomedical applications of PEG‐based materials and their key functions in various formats

PEG format	Biomedical applications
Bulk	Substrate, lubricant, and softener
Hydrogel	Therapeutic agents delivery, tissue engineering, cell culture, 3D bioprinting, bone regeneration, wound healing, and wearable biosensor
Thin solid film	Antibacterial dressing and surface coating of medical devices
Nanoparticle	Therapeutic agents delivery and bioimaging

Of particular interest is PEG's chemical structure (H−(O−CH_2_−CH_2_)_n_−OH), providing it with an exceptional capability to encapsulate or covalently link hydrophobic drugs, giving them hydro‐solubility. Further, PEG has been proved to be equipped with high stability during internal body circulation,^[^ ^25^ ^]^ which benefits from its non‐toxic property and low immune elimination. With hydroxy (OH) serving as the terminal group, PEG can be used as a versatile building block that presents an advantageous capability to be chemically functionalized with a variety of targeting groups or responsive segments such as ortho ester, imine, ketal, and acetal. With judicious functionalization, it can become responsive to stimuli such as pH, temperature and redox reactions to release cargo drugs in the tumor's microenvironment when needed. Further, when PEG is used in the form of a water‐swelling hydrogel, the size of hydrogel nanoparticles can be precisely adjusted into a suitable range to achieve an enhanced permeability and retention (EPR) effect.^[^ ^24^ ^]^ Altogether, these pivotal features have made PEG‐based materials attractive to develop therapeutic strategies for localized and on‐demand drug delivery.

In PEG‐hydrogel DDSs, drugs are typically encapsulated into the structure through non‐covalent entrapment, where they are locked within the matrix cross‐link, preventing the drug's unabridged structure from chemical bonding. Subsequently, as the responsive segments gradually break, the PEG network degrades and facilitates the release of the loaded drug without the elution of undesirable or toxic monomers. PEG is a desirable biomaterial in this area, and its in vivo metabolism and biosafety have been well verified.^[^ ^26^ ^]^ The metabolism of PEG‐based hydrogels is correlated with their molecular weight (MW), where PEG‐hydrogels with lower molecular weights demonstrate higher metabolism performance and vice versa.^[^ ^26^, ^27^ ^]^ In a recent in vivo study, using a radioactive labeling technique, it has been shown that PEG hydrogel species are almost completely excreted within 10 days after the hydrogel implantation.^[^ ^27^ ^]^

**Figure 1:**
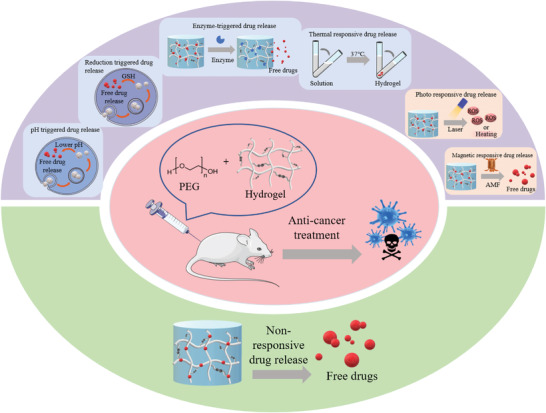
PEG‐based drug delivery systems for cancer treatment.

In this review article, we discuss advances and breakthroughs, reported in the past decade, on the development of PEG‐based hydrogels applied in anti‐tumor drug delivery. This review article addresses the merits of PEG hydrogels from various perspectives, including controlled drug release, biocompatibility, and their potential as multifunctional biomaterials. As illustrated in **Figure** **1**, we reviewed the progress in the development of PEG‐based DDSs categorized under stimuli‐responsive and non‐responsive drug release. The responsive drug delivery approaches cover the systems that function based on either exogenous stimuli‐response, such as photo‐ and magnetic‐sensitive PEG hydrogels, or endogenous stimuli‐response such as enzyme‐, pH‐, reduction‐, and temperature‐sensitive PEG hydrogels. Finally, the commercial potential of PEG‐based hydrogels in cancer therapy and the limitations that need to be addressed in future research for their clinical translation are highlighted.

## 2 Stimuli‐Responsive PEG‐Hydrogels for Cancer Therapy

### 2.1 Enzyme‐Sensitive PEG‐Hydrogels

Unlike normal tissues, cells in tumors secrete excessive enzymes that function like catalysts for cancer metastasis and invasion. Thus, the overexpressed enzymes can be used as a stimulus for the design of drug carriers that respond to tumor microenvironment.^[^ ^28^, ^29^, ^30^, ^31^ ^]^

In multicellular organisms, the extracellular matrix (ECM) is a non‐cellular component composed of various macromolecules and minerals present around the cells. The ECM is mainly composed of four kinds of substances, namely collagen, non‐collagen, elastin, proteoglycan, and aminoglycan.^[^ ^28^ ^]^ ECM plays a physical role in supporting cells, holding water, and connecting cells to form tissues and organs. It also plays an integral biological role in cells’ growth, death, polarity, shape, migration, and metabolic activities. ECM, and in particular extracellular collagen substrate, serves as the first barrier in the process of tumor metastasis.^[^ ^29^ ^]^ The degradation of ECM, thus, plays a key and beneficial role in aiding the invasion and metastasis of a tumor.

One of the typical examples of over‐produced enzymes in tumor cells is matrix metalloproteinases (MMP), a protease that targets and degrades many proteins present in the ECM. To promote the metastasis of tumor cells from the primary site to other sites, MMPs are overexpressed in the tumor microenvironment to degrade the ECM and extracellular collagen substrate. There are two main strategies for designing MMP‐sensitive nano‐drug carriers. One approach is to have the scaffolds of the nanocarriers enzymatic‐responsive, so they degrade with the presence of MMP and other fragments, including esters and carbamate polymers (peptides) that can be served as the cleavable site of MMP. Another strategy is that the drug carrier itself contains the structure of substrate for MMP, thus the drug carrier breaks down under the MMP environment.^[^ ^32^, ^33^, ^34^, ^35^ ^]^

One example of MMP‐sensitive PEG hydrogels is the work of Shen et al. who loaded such hydrogels with anti‐cancer drug doxorubicin (DOX) nanoparticles containing MMP‐degradable peptides and achieved a high drug targeted release efficiency.^[^ ^30^ ^]^ Fluorescent polystyrene nanoparticles and losartan, which were used to reduce collagen levels in tumor tissues, were encapsulated in the PEG‐hydrogel. Compared with a standard FPNP‐loaded hydrogel (with no losartan), the one that contained losartan resulted in enhanced penetration and reduced levels of collagen upon implantation.

**Figure 2:**
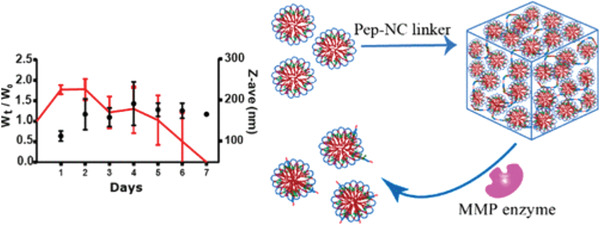
PEG‐hydrogel gradually degrades and releases the entrapped micelles under the existence of MMP enzyme. Reproduced with permission.^[^ ^32^ ^]^ Copyright 2020, American Chemical Society.

Among the MMP family, MMP‐2 and MMP‐9 are of particular importance since they can decompose fibrous collagen, the main component of ECM. Both Vermonden and Zhang's groups fabricated micelle‐based PEG‐hydrogels containing substrates for MMP‐2 or MMP‐9.^[^ ^31^, ^32^ ^]^ As shown in **Figure** **2**, the MMP‐sensitive PEG‐hydrogel breaks down in cancer tissue, releasing micelles that can be accessible to the intracellular tumor by endocytosis.^[^ ^32^ ^]^

Besides being the anti‐tumor drug carrier, MMP‐responsive PEG‐hydrogels can also deliver other therapeutics. Recently, for example, Erkoc et al. developed an MMP‐sensitive and peptide‐functionalized PEG‐hydrogel as the carrier of Quinacrine to increase apoptosis in brain cancer cells. Quinacrine is a recently discovered tumor necrosis factor *α*‐related apoptosis‐inducing ligand (TRAIL).^[^ ^33^ ^]^ This PEG‐hydrogel is fabricated by water‐in‐water emulsion polymerization to avoid the incompatibility between organic solvents or surfactants with the modified peptides. Further, Liu and other team members fabricated a smart photosensitizer delivered by a PEG‐hydrogel platform with a MMP‐sensitive property to achieve photodynamic therapy.^[^ ^34^ ^]^ The photosensitizer is released in a controlled manner by degrading the MMP‐cleavable sequence contained in the PEG‐hydrogel system. The PEG‐hydrogel developed by Shen et al. also contains losartan that can deplete collagen to enhance the penetration of drug carrier in cancer tissue.^[^ ^30^ ^]^

The second strategy to produce the MMP‐sensitive nanoparticles is based on a system that connects a drug molecule to a polymer skeleton using a linker as the substrate of MMP with the ability to decompose in the presence of a targeting enzyme. However, since drugs are easily entrapped into the 3D network structure of hydrogels, only a few studies have tried to covalently connect the drug with the PEG‐hydrogel carrier.

Apart from MMPs, other overexpressed enzymes, like elastase, are also applied as the stimuli in sensitive anti‐cancer materials.^[^ ^35^ ^]^ Only one work to date has been reported on PEG‐hydrogel DDSs that function based on their response to elastase. Relying on this concept, Wang et al. developed an enzyme‐stimulated nanogel, responsive to elastase, as a nanocarrier for drug delivery. It was shown that the nanogel has greater drug loading capacity with sustained DOX release properties triggered by elastase in comparison with the case of free DOX.^[^ ^36^ ^]^ The functionalization of PEG‐hydrogel with a fragment that could be cleaved by enzymes, such as elastase, is a promising route for enhancing the responsive drug release rate.

### 2.2 Magnetic Field‐Sensitive PEG‐Hydrogels

Strategies based on responses to magnetic fields have been universally applied in tumor targeting and imaging for a long period. Compared with the strategies based on endogenous stimulus‐response (pH, redox, and enzyme), magnetic responsiveness can be operated remotely through Magnetic Resonance Imaging (MRI) to achieve real‐time drug delivery to targeted tissues. Superparamagnetic iron oxide nanoparticles (SPIONs) are commonly used in magnetic response strategies. SPIONs generally contain Fe_2_O_3_, Fe_3_O_4_, and other ferrites with favorable properties such as small particle size, low toxicity, and biocompatibility.

However, one of the main challenges in the application of SPIONS is their hydrophobic character, making them prone to aggregation. This overshadows their biocompatibility and circulation stability. Another challenge is that a large range of effective anti‐cancer drugs, such as DOX and Vinorelbine,^[^ ^37^ ^]^ are water‐soluble, limiting the applicability of hydrophobic SPIONs in drug delivery. To solve this problem, SPIONs have been combined with other hydrophilic biomaterials, in particular PEG‐hydrogels.^[^ ^38^ ^]^

Magnetic hydrogels (MagGel) are a group of composite materials with an inorganic/organic hybrid structure composed of inorganic magnetic nanomaterials, such as Fe_3_O_4_ and organic hydrogels. MagGel benefits from the properties of both magnetic materials and hydrogels and shows outstanding biocompatibility and magnetic responsiveness. To increase their biocompatibility and circulation stability, polymers like PEG are typically used for the coating of their surfaces. Thus, PEG‐MagGel has been used as an environmentally sensitive anti‐tumor drug carrier for magnetically targeted delivery, magnetic responsive release, and magnetic hyperthermia.^[^ ^39^, ^40^ ^]^

The magnetic‐controlled drug release in a hydrogel drug carrier can be achieved either through an ON/OFF mechanism or based on a magnetocaloric effect.^[^ ^41^ ^]^The ON/OFF mechanism relies on the controlled change of the exogenous magnetic field. In the magnetocaloric effect, also referred to as the magneto‐thermodynamic phenomenon,^[^ ^42^ ^]^ under an alternating magnetic field (AMF), the change in the temperature of magnetic nanoparticles in MagGel increases the temperature of the surrounding gel matrix. The generated heat causes a structural change, such as swelling and contraction, in the thermal‐sensitive hydrogels and increases the drug release rate.

Based on the merits of PEG‐MagGel, M. Jaymand and coworkers have recently designed a stimulus‐responsive magnetite nano hydrogel (MNHG) with a remarkable magnetization value.^[^ ^42^ ^]^ Compared with traditional inorganic magnetic nanoparticles, the PEG‐hydrogels nanoparticles not only show superior biocompatibility and stability, but also are with higher drug loading capacity efficiency due to the interactions between PEG‐hydrogel functional groups and drugs by covalent or non‐covalent bonds, such as hydrogen and electrostatic bonding. Combining the thermal‐ and pH‐sensitivity of the polymeric carriers, these types of magnetite nano hydrogels could be considered as a smart DDS. However, the magnetization value of MNHG has not been comprehensively exploited, and the unavoidable cytotoxicity of magnetite needs to be carefully considered for any clinical application.

**Figure 3:**
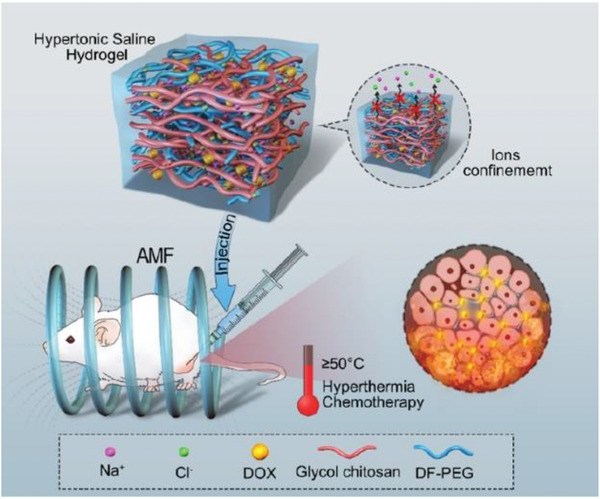
Nonmagnetic hypertonic saline‐based PEG‐hydrogel achieves both magnetic hyperthermia and pH‐sensitive chemotherapy in the MCF‐7 tumor‐bearing mice model. Reproduced with permission.^[^ ^43^ ^]^ Copyright 2019, American Chemical Society.

In order to overcome these drawbacks and maximize the benefits of the magnetic property, modified nonmagnetic PEG‐hydrogels have been developed as alternatives for traditional magnetite nanogels.^[^ ^43^ ^]^ To boost the therapeutic effect, it is necessary to make use of magnetic hyperthermia. Gao et al., for example, have recently developed a nonmagnetic hypertonic saline‐based PEG‐hydrogel to prevent postsurgical relapse of breast cancer, which produces sufficient heat under AMF to release the anti‐tumor drug in the desirable sites (**Figure** **3**).^[^ ^43^ ^]^ Besides, according to the Donnan equilibrium theory, the H^+^ ions may decrease the swelling ratio and lead to the collapse of the hydrogel, which means this platform can realize dual responsiveness (pH and magnetic field). This work is an exemplar of an approach that integrates magnetic hyperthermia and pH‐sensitive chemotherapy to reduce or even eliminate the possibility of postsurgical recurrence.^[^ ^44^ ^]^

### 2.3 Reduction‐Sensitive PEG‐Hydrogels

Reduction‐sensitive polymers mostly contain disulfide bonds (S—S) in their structure. The disulfide bond maintains strong stability under body temperature (37 °C) and physiological pH as well as in healthy tissue, while it breaks down in the presence of reductants such as glutathione (GSH) and dithiothreitol (DTT).^[^ ^45^ ^]^ In general, the S—S is reduced to sulfhydryl (‐SH), resulting in polymer degradation in aqueous media.^[^ ^46^ ^]^

Among the common reductants, GSH is one of the most abundant low molecular weight biological mercaptans in organisms. GSH and the oxidized glutathione (GSSG) constitute an essential redox couple in the microenvironment of living biological cells. GSH becomes enriched inside the cells due to the presence of GSH reductase, thus maintaining the intracellular reduction potential.^[^ ^47^ ^]^ In contrast, high oxidation potentials are present in the extracellular microenvironment because of specific extracellular proteins, such as glutathione peroxidase, that can stabilize GSSG in body fluids, extracellular matrix, and cell surface.^[^ ^48^ ^]^ Therefore, the redox potential difference between intracellular and extracellular microenvironment can provide favorable conditions for biological carriers to deliver therapeutical agents, such as pharmaceutical molecules,^[^ ^49^ ^]^ genes,^[^ ^50^ ^]^ and antigens.^[^ ^51^ ^]^

The intracellular environment in tumor tissues exhibits stronger reducibility than that of normal tissues.^[^ ^52^ ^]^ Consequently, reduction‐responsive polymers are favorable platforms for releasing a drug exclusively in the tumor sites. Indeed, PEG‐hydrogels have been considered as a reduction‐responsive carrier in recent decades due to their ease of modification by disulfide bonds.^[^ ^53^ ^]^ The modified disulfide bonds in PEG‐hydrogel can be reduced to sulfhydryl groups under the existence of essentially any reducing agent, thereby resulting in the rapid degradation of hydrogel and the release of loaded therapeutic agents.^[^ ^54^ ^]^

**Figure 4:**
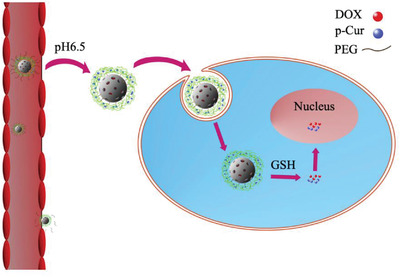
Illustration of the dual responsive strategies. This drug delivery system shows both redox and pH responsiveness. The PEG shell can break down under a weak acidic environment (pH 6.5), and then the drug is released under the presence of glutathione (GSH).

Building on this mechanism, Wang's group has recently developed pH and redox dual‐stage responsive nanocarriers by using PEG‐hydrogel as the main component of the carriers to achieve codelivery of curcumin and DOX.^[^ ^55^ ^]^ As illustrated in **Figure** **4**, the PEG shells are initially cleaved in the weak acidic condition in tumor tissue due to the presence of a pH‐sensitive benzoic—imine bond.^[^ ^55^ ^]^ The cationic hydrogel coating improves cell uptake. Subsequently, due to the redox effect of GSH, the hydrogel coating in tumor cells can be cleaved. Long‐lasting systematic circulation and high drug entrapment quantity can be achieved using such PEG‐based nanocarriers.

Apart from traditional chemotherapy, DDSs can be coupled with immunotherapy to achieve more effective cancer treatment. In the work of Kapadia et al., for example, the advantage of PEG‐hydrogel as the antigen and immunostimulatory adjuvant carrier is well demonstrated.^[^ ^56^ ^]^ PEG‐hydrogel was easily modified with disulfide bonds as reduction‐responsive cleavable linkers. In addition, the introduction of amine groups kept the positive charge of the whole nanoparticle, which in turn increased the cellular uptake and intracellular release of the antigenic peptide. The immunostimulatory adjuvant carried in this system can enhance the proliferation of potent antigen‐specific T cell and strengthen the immunity.

### 2.4 Photo‐Sensitive PEG‐Hydrogels

Photo responsiveness has been one of the most effective and widely studied exogenous response strategies for on‐demand drug delivery. UV ray, visible light, and near‐infrared (NIR) light are typically used as exogenous stimuli. Numerous studies have designed smart PEG‐hydrogels that deliver photosensitizers to target sites and enhance therapeutic efficacy. Two strategies have been utilized in this domain: i) hydrogel‐loaded chemotherapeutics, such as anti‐cancer drugs, that are released by illumination, and ii) photosensitizers, such as azobenzene, astragalus, and triphenylmethane, that are incorporated in DDS therapeutic platforms through chemical conjugation or physical encapsulation. Under the stimulation of an external laser, the photosensitizers generate reactive oxygen species (ROS) or heat to kill cancer cells. Upon the application of light with an appropriate wavelength to PEG‐hydrogels, that are modified with photosensitizers, the chromophore contained in the hydrogels changes its conformation through photoreaction.^[^ ^57^ ^]^ Photoreactive small molecules lead to structural changes in PEG‐hydrogels by photochemical reactions, for example, photo‐triggered decomposition, isomerization, cross‐linking, and degradation.

For the photo‐triggered DDS, the encapsulated anti‐cancer drug can be continuously released from the nanocarrier when a photo‐responsive structural change occurs. Recently, Zhao et al. constructed a photopolymerizable PEG‐hydrogel system based on this approach to avoid postoperative recurrence of glioblastoma,^[^ ^58^ ^]^ one of the most common primary malignant brain tumors. The authors showed that the injection of the photopolymerizable hydrogel into the tumor resection cavity of a mouse model was effective in sustaining the release of the anti‐cancer drug paclitaxel (PTX) encapsulated in PLGA nanoparticles. It was demonstrated that this strategy prevents the recurrence of glioblastoma multiforme (GBM).

**Figure 5:**
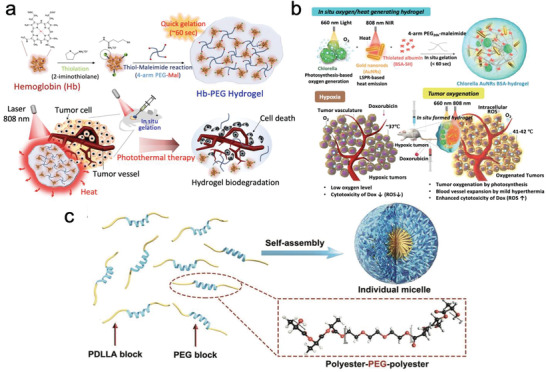
a) In situ oxygen‐ and heat‐generating anti‐cancer hydrogel.^[^ ^60^ ^]^ Reproduced with permission.^[^ ^60^ ^]^ Copyright 2019, Elsevier. b) NIR light‐responsive heat‐emitting biodegradable hydrogel. Reproduced with permission.^[^ ^61^ ^]^ Copyright 2019, Elsevier. c) Sprayable PTT PEG‐hydrogel for postsurgical treatment of cancer. Reproduced with permission under the terms of the Creative Commons license.^[^ ^62^ ^]^ Copyright 2018, the Authors. Published by Wiley‐VCH GmbH.

Photodynamic therapy (PDT) and photothermal therapy (PTT) are the other two main approaches employed in photo‐responsive therapy. PDT refers to a process in which tumor cells intake the nanodrugs with a photosensitizer and thereby experience a photochemical reaction under light irradiation at a specific wavelength. This process results in the generation of cytotoxic oxygen‐free radicals or singlet oxygen.^[^ ^59^ ^]^ Similarly, intense heat is generated during the PTT process. This process results in the injury of the tumor cell's membrane and vascular endothelial cells in the tumor sites, thus killing the cancer cells (**Figure** **5**).

One of the recent works in this area is reported by Youn et al. who developed a dual‐functional, ROS‐generating, and heat‐emitting therapeutic PEG‐hydrogel platform to combat breast cancer cells (Figure 5a).^[^ ^60^ ^]^ This platform contains chlorella and gold nanorods, which in response to near‐infrared (NIR) radiation, generate ROS and heat to enhance cytotoxicity in the tumor tissue. Experiments in vitro and in vivo have been conducted, demonstrating that this system is effective in killing 4 T1 breast cancer cells. Another work of interest in this area is a bio‐secure PTT system that is based on hemoglobin PEG‐hydrogel and is equipped with a NIR‐responsive heat‐generating property (Figure 5b).^[^ ^61^ ^]^ A hemoglobin (Hb) hydrogel, a natural substance from the human body, was used in this work. This system showed promising treatment against A549 lung cancer cells both in vitro and in vivo when stimulated using 808 nm laser.

In another work, to improve the practical value of the designed healing hydrogel, Yu's group designed sprayable PTT hydrogel based on black‐Phosphorus nanosheets for postsurgical therapy (Figure 5c).^[^ ^62^ ^]^ In this work, the favorable biodegradability and biocompatibility of the PPT‐DDS system were demonstrated using both in vitro and in vivo experiments, showing that the sprayable hydrogel can significantly eliminate the residual Hela cells, a common human cervical cancer cell line.

To track the therapy route, most of the drug‐loaded nanoparticles can also be used as fluorescent imaging agents with key roles in image‐guided therapy and therapeutic monitoring. For example, Wang et al. created an MMP‐intelligent targeted PDT system formed on oligo(p‑phenylenevinylene) derivative PEG‐hydrogel. This hybrid hydrogel can be used as a photosensitizer to generate ROS upon light illumination and serve as a platform for tumor‐specific imaging and killing.^[^ ^34^ ^]^

**Figure 6:**
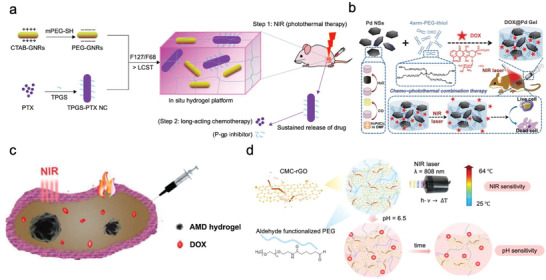
a) A dose‐adjustable in situ hydrogel platform based on PEG‐gold nanorods. Reproduced with permission.^[^ ^63^ ^]^ Copyright 2018, Elsevier. b) Nanosheet‐knotted injectable PEG‐hydrogels. Reproduced with permission.^[^ ^64^ ^]^ Copyright 2020, Royal Society of Chemistry. c) Injectable dual‐functional PEG‐hydrogel used for both PTT and chemotherapy. Reproduced with permission.^[^ ^65^ ^]^ Copyright 2018, Elsevier. d) PTT hydrogel and a pH‐triggered drug release system for the treatment of cancer. Reproduced with permission.^[^ ^66^ ^]^ Copyright 2017, Elsevier.

Notably, a novel synergistic chemo‐photothermal therapy has recently been introduced as one of the most promising and efficient anti‐tumor treatments (**Figure** **6**). Gao et al. developed a local therapeutic PEG‐hydrogel platform combining PTT with long‐acting chemotherapy for the treatment of colorectal cancer, particularly when multidrug resistance occurs. This hybrid platform consists of (PEG)‐coated gold nanorods and D‐alpha‐tocopheryl PEG 1000 succinate‐coated paclitaxel (PTX) nanocrystals, all incorporated into an in situ hydrogel system for injection (Figure 6a). In this system, heat is generated by the NIR‐responsiveness of gold nanorods to shrink tumor followed by long‐acting chemotherapy achieved by the long‐lasting release of PTX together with P‐glycoprotein inhibitor TPGS to reverse the drug resistance.^[^ ^63^ ^]^

In two other related works in this area, Lu et al. (Figure 6b) and Chen et al. (Figure 6c) combined PTT with drug‐release action to form a double‐functional therapy system in which the emitted heat was able to facilitate the anti‐cancer drug release.^[^ ^64^, ^65^ ^]^ Liu's group developed PEG‐hydrogels containing palladium nanosheets with NIR‐responsive and heat‐generating properties. Combined with chemotherapy via the encapsulation of DOX, the entire system exhibits pronounced cytotoxicity in breast cancer cells (4T1 cells) as evidenced by both in vitro and in vivo results. The in vivo experiment in 4T1 tumor‐bearing mice provided evidence for decreases in tumor volume after treatment by DOX@Pd Gel + laser. Chen's team developed MoS_2_/Bi_2_S_3_‐PEG‐hydrogels@DOX with photothermal transformation ability and chemotherapy, demonstrating a significant anti‐cancer effect against HT29 xenografted tumor in vivo and in vitro. In another work, Shang et al. established a dual‐platform equipped with PTT and pH‐responsive drug release performance to achieve targeted release of anti‐tumor drug (DOX) and heat (Figure 6d).^[^ ^66^ ^]^ In this research, the authors developed a reduced graphene oxide (rGO) hybridized hydrogel as a near‐infrared (NIR)/pH dual‐responsive platform for combined chemo‐photothermal therapy. Carboxymethyl chitosan‐functionalized rGO/aldehyde functionalized PEG‐hydrogels with photothermal performance and pH responsiveness were fabricated with the ability to degrade into CMC, CHO‐PEG, and GO under acidic conditions. Due to the degradability of dynamic Schiff base linkages used in this system, the DOX release from the rGO‐PEG hydrogel was greater in the cancerous biological environment than in the physiological environment. This system showed favorable biocompatibility and low cytotoxicity in normal tissue (L‐929) cells.

In summary, multifunctional photo‐responsive nanoparticles hold great potential to improve anti‐cancer efficacy and reduce adverse reactions to cancer phototherapy. Further, synergistic chemo‐photothermal therapy has attracted particular attention, and it is a high possibility that synergistic therapy will take the dominant place in the future of cancer treatment.

### 2.5 Thermo‐Sensitive PEG‐Hydrogels

Temperature change is utilized as the exogenous stimuli in thermo‐responsive PEG hydrogels to control their drug release behavior.^[^ ^67^ ^]^ Hydrogel has a hydrophilic, 3D network structure, which can maintain a swelling‐state with a large volume of water inside. Generally, as a widely used system for tissue engineering and drug delivery, thermo‐sensitive hydrogels form a 3D network structure via sol–gel transformation at the body temperature.^[^ ^68^ ^]^

Tumor and inflammatory tissues are often more hypothermic compared with normal tissues. Thus, a series of methods, including magnetic induction, ultrasound, and hot water bath, are used to heat the tumor site, to increase the blood flow and vascular permeability inside the tumor. This can lead to the accumulation of anti‐cancer drugs inside the tumor tissue and thus promoting the apoptosis of cancer cells. Based on this mechanism, temperature‐sensitive nanocarriers are widely investigated as the building block of targeted nanocarriers. Importantly, thermo‐sensitive hydrogels, show temperature‐triggered solubility (gel to sol) and therefore enable the release of therapeutic agents in a controlled manner. Thermo‐responsive polymers, for example, poly N‐isopropylacrylamide (PNIPAAM),^[^ ^69^ ^]^ polyvinyl alcohol‐vinyl acetate copolymer (PVA),^[^ ^70^ ^]^ and polyvinylpyrrolidone (PVP),^[^ ^71^ ^]^ have been typically used to create hydrogel‐based, thermo‐sensitive DDSs.

**Figure 7:**
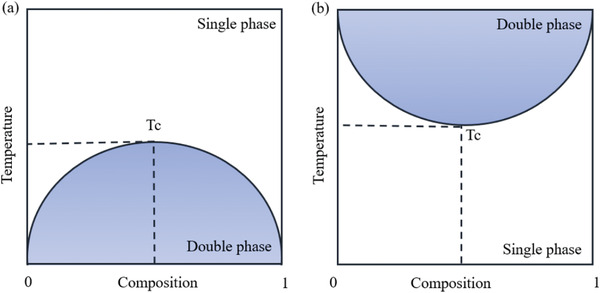
Typical diphase diagrams of polymer solutions with a) UCST and b) LCST properties.

Thermo‐sensitive polymers can be divided into Upper Critical Solution Temperature (UCST) polymers and Lower Critical Solution Temperature (LCST) polymers according to the different transformation behaviors at Tc (**Figure** **7**).^[^ ^72^, ^73^, ^74^ ^]^ As shown in Figure 7a, for a UCST‐type polymer solution system, when the temperature is higher than Tc, the polymer solution clarifies and presents a homogeneous phase, while for temperatures below Tc, solid–liquid separation occurs between the polymer and solvent, presenting a cloudy solid–liquid phase (Figure 7a).^[^ ^75^, ^76^ ^]^ For an LCST‐type polymer solution system (Figure 7b), the polymer dissolves below a critical solution temperature (Tc), above which phase separation occurs in the polymer solution.

**Figure 8:**
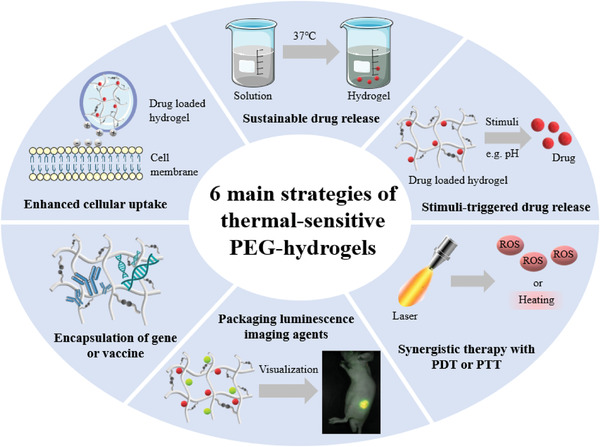
Six main strategies utilized for the application of thermal responsive PEG‐hydrogel in cancer therapy.

The main drug‐release strategy in thermo‐sensitive PEG‐hydrogels relies on the phase transition property of LCST polymers, as they are transformed into gel at the body temperature (37 °C). Such a gel delivery system is thus capable of simulating the human extra cellular matrix and providing sustainable release of the loaded drug.^[^ ^77^ ^]^ According to the specific design of the therapeutic platform, the research works around thermosensitive PEG hydrogels for cancer treatment can be classified into six sub‐groups, as schematically shown in **Figure** **8**.

Thermosensitive PEG‐hydrogels can provide sustainable drug release. For example, Li and his group members developed a responsive PEG‐based thermo‐gelling system for liver cancer treatment, in which PEG played a significant role in keeping the thermal stability of the whole system above the normal body temperature.^[^ ^78^ ^]^ Similarly, several studies have proved that thermosensitive PEG‐hydrogels perform well in continuously releasing anti‐tumor drugs, like DOX,^[^ ^79^, ^80^ ^]^ paclitaxel (PTX),^[^ ^81^, ^82^ ^]^ cisplatin (DDP),^[^ ^82^ ^]^ and dimethoxy curcumin.^[^ ^83^ ^]^ Besides the anti‐cancer drugs, cytokine regulatory substances can also be added to the delivery system. For instance, Tsai et al. added bevacizumab to the system, which can prevent the expression of vascular endothelial growth factor (VEGF) to stop the growth of tumor tissues.^[^ ^84^ ^]^ Also, Johnson's lab has created a copolymer‐based injectable hydrogel that contains PTX and resiquimod to up‐regulate tumor necrosis factor TNF‐*α*.^[^ ^85^ ^]^ In addition, hydrogel‐based supramolecular assemblies have also been used as a DOX‐encapsulated platform.^[^ ^86^ ^]^

**Figure 9:**
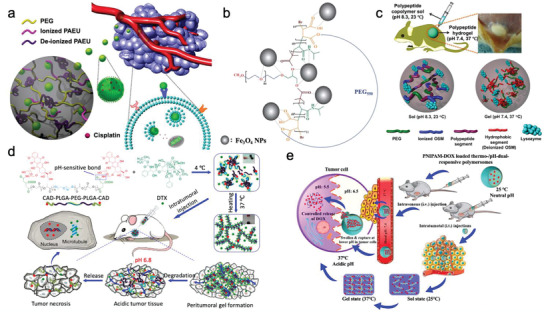
Thermal responsive PEG‐hydrogel with controlled drug release property. a) A temperature‐sensitive PEG‐hydrogel to pH‐dependently release DDP. Reproduced with permission.^[^ ^87^ ^]^ Copyright 2017, Royal Society of Chemistry. b) A pH‐triggered degradable magnetite PEG‐hydrogel with high encapsulation and DOX release efficiencies. Reproduced with permission.^[^ ^42^ ^]^ Copyright 2018, Wiley Periodicals LLC. c) A pH‐sensitive therapeutic protein delivery system. Reproduced with permission.^[^ ^88^ ^]^ Copyright 2018, Royal Society of Chemistry. d) An injectable thermosensitive PEG‐thermogel with pH‐triggered cleavage property. Reproduced with permission.^[^ ^89^ ^]^ Copyright 2018, Elsevier. e) A dual stimuli‐responsive injectable hydrogel delivery system. Reproduced with permission.^[^ ^90^ ^]^ Copyright 2018, Elsevier.

Another type of PEG‐hydrogel DDS functions based on both thermosensitive phase transition and stimuli‐triggered drug release properties. Notably, pH has become a widely accepted stimulus because of significant differences in pH values of intracellular and extracellular environments. Recent research works that have tested the efficiency of pH‐triggered drug release using a thermosensitive PEG hydrogel are schematically summarized in **Figure** **9**.^[^ ^42^, ^87^, ^88^, ^89^, ^90^ ^]^

To achieve another dimension of control in functionality, thermosensitive phase transition can be combined with a magnetic‐triggered property. An example is the work of Zhao et al. who proposed a magnetic hydrogel showing controlled drug release in response to surrounding magnetic fields.^[^ ^91^ ^]^ Doped with Fe_3_O_4_, the magnetic field responsiveness of this system means that it can produce a large amount of heat to control the surrounding temperature under the alternative magnetic field (AMF).

To realize synergistic treatment of cancer, thermo‐sensitive PEG‐hydrogel systems can be combined with chemotherapy and other treatments, like photothermal therapy (PTT), photodynamic therapy (PDT), radiation therapy, and immunotherapy.^[^ ^92^, ^93^, ^94^, ^95^, ^96^, ^97^, ^98^ ^]^ Synergistic therapy has been widely used for the treatment of various types of cancer, holding great potential in future of cancer therapy. As listed in **Table** **3**, PEG‐based thermogel systems have been used in both chemotherapy and other therapy strategies like PTT, PDT, immunotherapy, and radiotherapy.

**Table 3:** Synergistic therapies using PEG‐thermogel systems

**Cancer Type**	**Chemotherapy Agents**	**Synergistic Therapy Strategy**	**Synergistic Therapeutic Compound**
Colon cancer	DOX	PTT	MoS_2_/Bi_2_S_3_‐PEG nanosheet^[^ ^92^ ^]^
Bladder cancer	DOX	PDT	Zinc phthalocyanine (ZnPC)^[^ ^93^ ^]^
Gastric cancer	PTX	PDT	Tetrandrine (Tet)^[^ ^94^ ^]^
Ovarian cancer	DOX	Immunotherapy	Inducible nitric oxide synthase (iNOS) substrate^[^ ^95^ ^]^
Melanoma	DOX	Immunotherapy	IL‐2 and IFN‐g^[^ ^96^ ^]^
Melanoma and liver cancer	DOX	Radiotherapy	Gold nanoparticles (AuNPs)^[^ ^97^ ^]^
	Gemcitabine derivative (GemC16)	Radiotherapy	Gemcitabine derivative (GemC16)^[^ ^98^ ^]^

**Figure 10:**
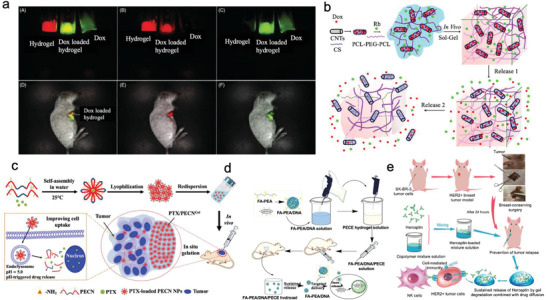
a,b) A drug delivery system based on PEG hydrogel capable of monitoring the therapeutic process. Reproduced with permission.^[^ ^99^, ^100^ ^]^ Copyright 2014, Elsevier, Copyright 2018, Elsevier. c) A positively charged thermo‐gel with easier endocytosis ability. Reproduced with permission.^[^ ^101^ ^]^ Copyright 2020, Royal Society of Chemistry. d) Thermo‐responsive PEG‐hydrogel for DNA delivery. Reproduced with permission.^[^ ^102^ ^]^ Copyright 2016, Springer Nature. e) An antibody delivery system based on PEG and PLGA co‐polymers to avoid postoperative recurrence of breast cancer. Reproduced with permission.^[^ ^103^ ^]^ Copyright 2019, National Center for Biotechnology Information.

PEG‐hydrogel nanomedicine materials can also achieve visualization of the therapy process when imaging agents are incorporated into their structure. As shown in **Figure** **10**a,b, Lv et al. have recently designed a thermosensitive hydrogel system with four‐arm PEG–PCL copolymer.^[^ ^99^ ^]^ For this system, porphyrin was used as the fluorescence dye, and the hydrogel showed dual function of fluorescence imaging and injectability in vivo. In another work, Lv et al. developed a programed PCL‐PEG‐PCL thermosensitive hydrogel. This system was combined with chitosan‐multiwalled carbon nanotubes with doxorubicin (DOX) and rhodamine B (RB) as model drugs. Using in vivo fluorescence imaging, it was shown that such co‐loaded dual drug delivery system could be tracked in real‐time.^[^ ^100^ ^]^

PEG‐based thermosensitive anti‐cancer drug delivery systems can be modified to achieve optimum penetration and/or targeting ability. For instance, Dong and coworkers created thermo‐sensitive PEG‐hydrogel based on triblock copolymers with small positive charges by introducing amino groups for better cell uptake efficiency (Figure 10c).^[^ ^101^ ^]^ The responding ability to the acidic environment of the endosome enhanced the release rate of the drug for up to 10 days.

The final carrier system reviewed in this section contains gene and vaccine vectors for cancer treatment. Figure 10d demonstrates a targeted delivery system of tumor suppressor DNA to kill cancer cells.^[^ ^102^ ^]^ Chen et al. created a delivery platform using injectable and thermosensitive PEG/PLGA hydrogels for Herceptin, a long‐acting HER2‐targeting antibody (Figure 10e).^[^ ^103^ ^]^ The authors showed that the degradation rate and drug release kinetics of the hydrogel‐based system can be feasibly tuned by changing the proportion of PEG and PLGA hydrogels. Through optimizing this proportion, the initial burst release of Herceptin was avoided, while a sustained release of this drug in vitro for up to 80 days was achieved. This duration is among the longest periods of Herceptin delivery reported in the literature.

In summary, PEG‐based hydrogels can release their cargo when the external temperature rises. The hybrid platforms developed based on this mechanism are of interest for heat‐triggered drug delivery considering their ability to remotely control the switch from an inert, cytocompatible state to a highly cytotoxic state, resulting from the release of on‐demand chemotherapeutic drugs.

### 2.6 pH‐Sensitive PEG‐Hydrogels

Employing the low pH of the cancer tissue environment as a stimulus has been well documented in the literature as a powerful and effective strategy for the design of DDSs.^[^ ^104^, ^105^ ^]^ The pH of the tumor extracellular, with an average value of 6.0–7.0 is lower than that of intracellular (7.4) due to the high glycolysis rate of cancer cells and the low oxygen conditions of the tumor.^[^ ^104^, ^105^ ^]^ The low extracellular pH value in the tumor leads to the progression of cancer from an in situ tumor, where the abnormal cells have not spread beyond where they first occurred, to metastatic cancer.^[^ ^106^ ^]^ In this regard, pH‐sensitive polymer materials can provide the required “intelligence” for targeted drug delivery. In recent years, hydrogel materials have been widely studied in this field as smart carriers for the delivery and controlled release of therapeutic agents. Once administered, the hydrogel exhibits a sol–gel phase transition by forming new chemical bonds or physically interacting with the operating environment.^[^ ^107^ ^]^ Among the hydrogels that can be used in this domain, those that are pH‐sensitive and exhibit phase changes in response to variations in pH are of special importance.^[^ ^108^, ^109^ ^]^

The key mechanism of delivery and release for the pH‐responsive hydrogels used as DDS systems is the swelling‐gel phase transition caused by the breakage of the chemical bonds due to their bespoke chemical structure.^[^ ^110^ ^]^ This can be achieved by introducing pH‐sensitive functional groups and chemical bonds into the molecular structure of hydrogels. Various covalent bonds, including amides, disulfides, thioethers, Schiff bases (imine,^[^ ^111^ ^]^ oximes and hydrazones) and click methods, such as azide‐alkyne cycloaddition, Diels–Alder reaction, Thiolene, and Michael additions have been applied in developing pH‐responsive hydrogels.^[^ ^112^, ^113^ ^]^

Another desirable property that can be achieved by the incorporation of suitable chemical functional groups and linkages is mechanical adaptability. Although hydrogels are extremely promising as biomaterials, their clinical application as a drug delivery reservoir requires material optimization in terms of ease of administration and patient compliance.^[^ ^1^ ^]^ The minimally invasive administration of drug‐eluting hydrogels requires that they have viscoelastic self‐healing ability to ensure reversible sol–gel transition during in vivo administration.^[^ ^2^, ^3^, ^4^ ^]^ It has been demonstrated that mechanical adaptability can be introduced into the matrix by using dynamic covalent bonds to make hydrogels.^[^ ^114^, ^115^ ^]^

In recent years, PEG has been widely used in chemical cross‐linking and end‐capping treatment processes as an ideal reagent to be functionalized with pH‐responsive groups to obtain block polymer hydrogels for targeted drug delivery systems^[^ ^116^, ^117^ ^]^ Methacrylic acid (MAA) is one the most widely used compounds in the development of pH‐sensitive hydrogels due to its biocompatibility and ease of copolymerization with other monomers.^[^ ^118^ ^]^ The incorporation of this acid into the hydrogel results in changes in the swelling performance of the hydrogel under high pH conditions due to the ionization of the carboxyl group. Ahmad et al., for example, harnessed this strategy to create a chemically cross‐linked PEG‐poly(methacrylic acid) oral hydrogel (PEGMA 4000) by free radical polymerization for pH‐responsive colon‐targeted administration of oxaliplatin (OXP).^[^ ^119^ ^]^ In another recent work, Lee et al. prepared CS‐Nanogels with PEG as a carrier between CS‐aminomalonic acid (CSAMA) conjugate and cisplatin (CDDP) through chelating ligand‐metal coordination for cancer cell‐specific delivery of CDDP.^[^ ^87^ ^]^

In another work relevant to this topic, Zhang et al. reported a pH‐sensitive hydrogel‐based system for oral delivery of 2‐ME.^[^ ^120^ ^]^ This work relies on encapsulating 2‐ME micelles with high anti‐tumor activity and low toxicity in pH‐responsive microspheres with a small particle size of 58 nm and high drug loading capacity (7.94 ± 0.23%). The in vivo imaging technology used in the animal experiments of this study shows that micellar microspheres are promising candidates for oral delivery of 2‐ME for cancer treatment. The results in this study support that 2‐ME‐micelle‐microspheres can prolong the residence time of drugs in mice.^[^ ^120^ ^]^ Another example is the work of Messersmith et al., who used Boric acid‐functionalized polyethylene glycol and polyphenols to fabricate bioactive injectable pH‐response hydrogels. The authors proposed a mechanism based on the dynamic properties of borate bonds, regulating the release of therapeutic polyphenols to the surrounding environment.^[^ ^121^ ^]^

In addition to acidic compounds, pyrimidine compounds are also attractive reagents for the preparation of pH‐responsive hydrogels. Sulfamethazine, for instance, is widely regarded as a suitable reagent for the preparation of anionic pH‐sensitive hydrogels. It is prone to sol–gel phase transition in the lower local pH range of 6.5–7.0, resulting in the formation of a solidified gel at the tumor site.^[^ ^122^, ^123^, ^124^ ^]^ Lee et al have made important contributions in this field. In 2016, they developed a new sulfamethazine‐based(OSM) anionic pH‐sensitive block copolymer hydrogel that can be used to prepare a radiopaque embolism material used for hepatic artery embolization chemotherapy.^[^ ^125^ ^]^ Similarly, based on this work, Lee et al. in 2018, once again reported a pH/temperature‐responsive comb‐shaped polypeptide block copolymer hydrogel composed of PEG and OSM for controlling protein delivery.^[^ ^88^ ^]^

Over the past few years, the UPy‐PEG hydrogel system has been extensively explored for cancer treatment.^[^ ^1^, ^2^, ^3^ ^]^ Due to the reversible interaction of the supramolecular material, the sol–gel transition occurs as a response to pH. Such switchable behavior is used to obtain an in situ gel drug depot that can be injected at pH 9.0 and forms a hydrogel when in contact with tissue at physiological pH.^[^ ^2^ ^]^ As one example, Dankers et al. recently proposed a cholesterol coupling strategy to increase lipophilicity to obtain a small molecule modified with MPC (mitomycin‐PEG24‐cholesterol) drug prepared by UPy‐PEG carrier, MMC (mitomycin C), and cholesterol. This new composite hydrogel system enables sustained release of small molecule hydrophilic drugs, such as mitomycin C, with important clinical significance for the treatment of Colorectal cancer (CRC).^[^ ^126^ ^]^

The swelling‐gel phase transition caused by pH response is the most basic feature of hydrogels used as DDSs. Although such DDSs are attractive because of their ease of handling and storage and the ability to avoid blocking long needles or catheters; they still have certain deficiencies. The most important shortcoming of these hydrogel systems is their poor cell absorption and slow drug release from endosomes,^[^ ^1^, ^2^ ^]^ leading to low target bioavailability and reduced therapeutic effect.^[^ ^3^, ^4^ ^]^ Further, it has been reported that the repeated injections of PEGylated nanocarriers can induce significant immune responses in animal models by inducing IgM antibodies,^[^ ^5^ ^]^ leading to their rapid clearance during repeated injections. This phenomenon is called accelerated blood clearance (ABC).^[^ ^6^, ^7^, ^8^ ^]^ In addition, clinical reports have been recorded on acute hypersensitivity reactions in some individuals after infusion of pegylated liposomes (such as Doxil).^[^ ^9^, ^10^ ^]^

In recent years, a wide variety of multi‐stimuli‐responsive hydrogels have been fabricated (pH/thermal, pH/magnetic, pH/redox, and pH/photothermal response), which not only keep the advantage of pH response in the DDS system but can potentially address the shortcomings mentioned above. One example out of many is a thermo‐pH responsive DDS fabricated by Alibolandi et al. who loaded heat‐responsive poly (N‐isopropylacrylamide)‐doxorubicin (PNIPAM‐DOX) into pH‐responsive polyethylene glycol‐2,4,6‐trimethoxybenzylidene pentaerythritol carbonate (PEG‐PTMBPEC) polymer. The new dual‐responsive hydrogen body system can significantly extend the cycle time of doxorubicin, enhance its accumulation in tumors (C26), and prolong its residence time in tumors, thereby significantly improving its therapeutic effect.^[^ ^90^ ^]^The in vivo results showed that such DDS significantly inhibits tumor growth rate in mice in comparison with free DOX delivery.

In another work, Zhao et al. prepared a drug‐loaded HTS‐polyethylene glycol injectable hydrogel to achieve pH/AMF dual stimulation response drug delivery and magnetothermal chemotherapy for postoperative breast cancer recurrence prevention. The HTS‐PEG dual stimulus responsive DDS can accelerate the rapid temperature rise of the mixed hydrogel formula with the help of an alternative magnetic field (Amf), which was called as magnetic‐mediated hyperthermia (MMH). This high‐temperature superconducting thermal therapy can promote the release of anti‐cancer drugs. Under this mechanism, the dipolar polarization of charged ions under high‐frequency electromagnetic radiation is confined to the biocompatible hydrogel, which helps prevent breast cancer recurrence after surgery.^[^ ^43^ ^]^ In another work, Su et al. fabricated a highly efficient carboxymethyl chitosan(CMC)‐modified reduced graphene oxide(rGO)/aldehyde functionalized poly (ethylene glycol) (CMC‐rGO/CHO‐PEG) hybridized hydrogel as an efficient near‐infrared (NIR)/pH dual‐responsive platform for combined chemo‐photothermal therapy. In the toxicity test experiments carried at pH 6.5, the amount of DOX released from the CMC‐rGO/CHO‐PEG system was reported to be close to 100% and much higher than that achieved under physiological environment (pH 7.4). These results, in particular, indicate that this system has great potential for DOX delivery and release under the acidic environment of tumor sites.^[^ ^66^ ^]^

In summary, pH‐responsive hydrogels are one of the most common types of DDS in cancer therapy applications, with quite mature drug delivery and release mechanisms and well‐defined hydrogel synthesis protocols. However, most hydrogels that encapsulate chemotherapeutic agents can only provide short‐term release profiles through passive encapsulation. This deficiency is because the pH‐sensitive chemical bonds are broken quite rapidly when stimulated by a change in pH. A strategy has yet to be developed to use in situ covalent interaction to control the release of the chemotherapeutic agent over durations in the range of a few months. Stimuli‐responsive PEG‐hydrogel systems used for cancer therapy are summarized in **Table** **4**.

**Table 4:** Stimuli‐responsive PEG‐hydrogel systems used for cancer therapy

Delivery system	Loaded drug	Responsiveness	Delivery target	Application	Drug release rate	In Vivo tumor growth inhibition (TGI)
FPNP/Los PEG^[^ ^30^ ^]^	DOX	Enzyme (MMP)‐sensitive	4T1 cells (murine breast cancer cell line)	Chemotherapy, enhanced penetration by losartan	100% within 10 h	59%
NanoDOX loaded^[^ ^31^ ^]^	DOX	Enzyme (MMP)‐sensitive	SCC‐15 cells (human squamous cell carcinomas cell line)	Chemotherapy	50% within 25 days	80%
HyMic^[^ ^32^ ^]^	Core cross‐linked (CCL) micelles	Enzyme (MMP)‐sensitive	HeLa cells (human cervical cancer cell line)	Cellular Internalization of CCL micelles by HeLa Cells	90% within 7 days	Not applicable
RGDS PEG^[^ ^33^ ^]^	Quinacrine	Enzyme (MMP)‐sensitive	U87 MG cells (Glioblastoma multiforme cell line)	Chemotherapy	80% within 3 days	Not applicable
PAMAM Dendrimers PEG^[^ ^36^ ^]^	DOX	Enzyme (MMP)‐sensitive	C6 cells (rat/rattus norvegicus glial tumor cell line)	Chemotherapy	50% within 38 h	Not applicable
RGD PEG^[^ ^34^ ^]^	OPVBT	Enzyme (MMP)/photo‐ sensitive	MCF‐7, MDA‐MB‐231 cells (human breast cancer cell line)	PDT, enhanced cell attachment and spreading	Not applicable	Not applicable
MNHG^[^ ^42^ ^]^	DOX	Magnetic field/pH‐sensitive	Not applicable	Chemotherapy	60% within 350 h	Not applicable
HTS‐PEG^[^ ^43^ ^]^	DOX	Magnetic field/pH‐sensitive	MCF‐7, 4T1 cells (human and murine breast cancer cell line)	Hyperthermia, chemotherapy	60% within 12 h	≈100% for MCF‐7, 85% for 4T1
MSNs PEG^[^ ^55^ ^]^	Phosphorylated curcumin, DOX	Reduction/pH‐sensitive	HeLa cells (human cervical cancer cell line)	Chemotherapy	80% within 25 h for phosphorylated curcumin, 70% within 25 h for DOX	Not applicable
PRINT®^[^ ^56^ ^]^	Antigenic peptide, immunostimulatory adjuvant	Reduction‐sensitive	T cells	Cancer vaccine, enhance cellular uptake	Not applicable	Not applicable
PEG‐DMA^[^ ^58^ ^]^	PTX	Photo‐sensitive	U87 MG cells (Glioblastoma multiforme cell line)	Post‐resection treatment	30% within 6 days	Not applicable (survival time enhanced)
BSA‐PEG^[^ ^60^ ^]^	Chlorella, gold nanorods	Photo‐sensitive	4T1 cells (murine breast cancer cell line)	Hyperthermia, tumor oxygenation, synergistic chemotherapy	Not applicable	≈100%
BP@PLEL^[^ ^62^ ^]^	Not applicable	Photo‐sensitive	HeLa cells (human cervical cancer cell line)	Photothermal therapy, postoperative treatment of cancer	Not applicable	≈100%
PEG‐GNRs^[^ ^63^ ^]^	PTX, gold nanorods	Photo‐sensitive	SW620 cells (human colorectal cancer cell line)	PTT, chemotherapy	45% within 144 h	≈100%
Palladium nanosheet PEG^[^ ^64^ ^]^	DOX	Photo‐sensitive	4T1 cells (murine breast cancer cell line)	PTT, chemotherapy	11 µg within 30 min	≈100%
PPDl‐PPG‐PEG^[^ ^78^ ^]^	DOX, docetaxel	Thermo‐sensitive	Hepatoma cells	Chemotherapy	40% within 13 days	≈100%
PLGA‐PEG‐PLGA^[^ ^79^ ^]^	5‐Fluorouracil, Chrysin	Thermo‐sensitive	HT29 cells (human colon cancer cell line)	Enhanced chemotherapy	100% within 100 h for 5‐fluorouracil, 90% within 100 h for chrysin	Not applicable
PLGA‐PEG‐PLGA^[^ ^80^ ^]^	DOX	Thermo‐sensitive	Saos‐2 cells (human osteosarcoma cell line)	Chemotherapy	60% within 15 days	93%
BSA‐PEG^[^ ^81^ ^]^	PTX	Thermo‐sensitive	MKN45 cells (human gastric tumor cell line)	Chemotherapy	30% within 144 h	73%
PEG‐PCL‐PEG^[^ ^82^ ^]^	PTX	Thermo‐sensitive	HeLa cells (human cervical cancer cell line)	Chemotherapy	Not applicable	≈50%
DiMC‐PEG^[^ ^83^ ^]^	Dimethoxycurcumin	Thermo‐sensitive	CWR22RV1 cells (human prostate cancer cell line)	Chemotherapy	80% within 120 min	Not applicable
PDLLA‐PEG‐PDLLA^[^ ^84^ ^]^	Bevacizumab, DOX	Thermo/pH‐sensitive	HeLa cells (human cervical cancer cell line)	Enhanced chemotherapy	73.56% for Bevacizumab, 61.21% for DOX within 36 days	87%
PLA‐PEG‐PNIPAM^[^ ^85^ ^]^	PTX, SN‐38, R848, gemcitabine	Thermo‐sensitive	CT26 cells (murine colorectal carcinoma cell line)	Chemotherapy, immunotherapy	100% within 15 days	≈100%
PNIPAAm‐PEG^[^ ^86^ ^]^	DOX	Thermo‐sensitive	AT3B‐1 cells (MDR cancer cells)	Chemotherapy	80% within 5 days	Not applicable
PEG–PAEUs^[^ ^87^ ^]^	Cisplatin	Thermo‐sensitive	A549 cells (human lung cancer cell line)	Chemotherapy	90% within 15 days	Not applicable
PEG‐PBLG‐OSM^[^ ^88^ ^]^	Lysozyme	Thermo/pH‐sensitive	Not applicable	Not applicable	≈250 ng mL^−1^ lasts for more than a week	Not applicable
PLGA–PEG–PLGA^[^ ^89^ ^]^	DOX, docetaxel	Thermo‐sensitive	H22 cells (mouse hepatocellular carcinoma cell line)	Chemotherapy	70% within 30 days	≈80%
PNIPAM‐PEG^[^ ^90^ ^]^	DOX	Thermo/pH‐sensitive	C26 cells (mice adenocarcinoma cell line)	Chemotherapy	60%‐80% within 144 h	≈83%
DF‐PEG‐DF^[^ ^91^ ^]^	DOX, DTX, Fe3O4	Thermo‐sensitive	L929 cells (Mouse fibroblast cell line)	Chemotherapy, magnetic hyperthermia	80% within 30 days	Not applicable
POR–PEG–PCL^[^ ^99^ ^]^	porphyrin	Thermo‐sensitive	Not applicable	In situ monitoring	Not applicable	Not applicable
CS/MBP/DOX^[^ ^92^ ^]^	DOX, Mos2/Bi2S3‐PEG	Thermo‐sensitive	L929 cells (Mouse fibroblast cell line)	Chemotherapy, PTT	20% within 48 h	≈75%
PCL‐PTSUO‐PEG^[^ ^93^ ^]^	DOX, ZnPc	Thermo‐sensitive	5637 cell (human bladder cancer cell line)	Chemotherapy, PDT	5.3% ± 2.1% for DOX, 95.2% ± 1.5% for ZnPc within 35 days	87.50%
PEG‐b‐PCL^[^ ^94^ ^]^	PTX, Tet	Thermo‐sensitive	BGC‐823 cells, SGC‐7901cells (human low‐differentiated gastric adenocarcinoma cell line)	Chemotherapy, PDT	50% within 5 days; 50% within 5 days	≈70%
PLGA‐PEG‐PLGA^[^ ^95^ ^]^	DOX	Thermo‐sensitive	4T1 cells (murine breast cancer cell line)	Chemotherapy, immunotherapy	90% within 10 days	86.62%
PELG‐PEG‐PELG^[^ ^96^ ^]^	DOX	Thermo‐sensitive	B16F10 cells (Mouse melanoma B16F10 cells with high metastatic cells)	Chemotherapy, immunotherapy	≈40% within 15 days	91.51%
Au‐DOX‐Ge^l[^ ^97^ ^]^	DOX,AuNPs	Thermo‐sensitive	B16 cells (mouse melanoma), HepG2 cells (Human hepatocellular liver carcinoma)	Chemotherapy, radiotherapy	15.7% within 24 h for DOX; 35.8% for AuNPs	64.60%
PLGA‐PEG‐PLGA^[^ ^98^ ^]^	Gemcitabine derivative (GemC16)	Thermo‐sensitive	4T1 cells (murine breast cancer cell line), B16F10 cells (Mouse melanoma B16F10 cells with high metastatic cells)	Chemotherapy, radiotherapy	65 within 19 days	≈80%
PCL–PEG–PCL^[^ ^100^ ^]^	DOX,RB	Thermo‐sensitive	BEL‐7402 cells (human liver cancer cell line)	Chemotherapy	20% for DOX and 50% for RB within 25 h	Not applicable
PCN‐b‐PEG‐b‐PCN^[^ ^101^ ^]^	PTX	Thermo‐sensitive	4T1 cells (murine breast cancer cell line)	Chemotherapy	50% within 48 h	94.27%

## 3 Non‐Responsive PEG‐Hydrogels for Cancer Therapy

As discussed in the previous section, PEG‐based responsive hydrogels are with excellent capabilities to overcome many difficulties associated with traditional anti‐cancer drugs, such as poor chemotherapy effects, systematic toxicity, and harmful side effects. However, there remain significant shortcomings in the application of stimuli‐responsive hydrogels in the controlled release of anti‐cancer drugs.

For example, when stimuli‐responsive hydrogels are used to deliver certain types of drug molecules, such as ciprofloxacin, angiotensin‐converting‐enzyme inhibitors benazepril (brand name, Lotensin), and enalapril (Vasotec), it is difficult to achieve suitable therapeutic effects because such molecules are only functional when present within the cells.^[^ ^127^ ^]^ Further, factors such as biocompatibility and biodegradability, response speed, sensitivity and precision, and mechanical strength of the 3D structure can be limiting parameters that restrict the widespread use of stimulus‐responsive hydrogels in clinical practice. Delivery of drugs from stimulus‐responsive hydrogels through the mechanisms such as gating, swelling, and disassembly response may also cause slippage or sudden release of the drug during administration. This may consequently reduce the efficacy of the drug delivery and release.^[^ ^127^, ^128^ ^]^ Therefore, only a few types of responsive hydrogels have so far succeeded to clinical applications. In contrast, many non‐responsive hydrogels are currently applied in the clinic via traditional administration methods, including oral administration,^[^ ^129^ ^]^ transdermal administration,^[^ ^121^ ^]^ and local injection administration.^[^ ^130^, ^131^ ^]^

**Figure 11:**
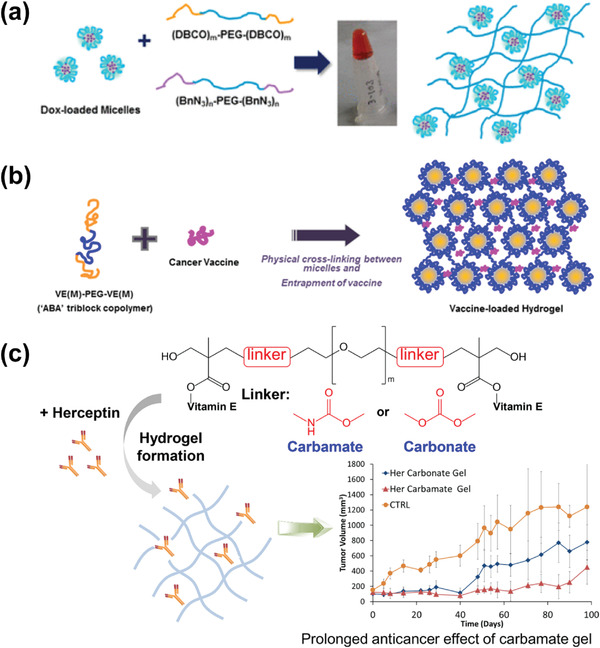
Schematic illustrations showing the formation of three “ABA”three‐stage PEG‐based hydrogels:a) Dox‐loaded PEG”ABA” hydrogel; b)Vaccine‐loaded “ABA” hydrogel; c) vitamin E‐PEG‐vitamin E triblock”ABA” hydrogel. Reproduced with permission.^[^ ^132^, ^133^, ^134^ ^]^ Copyright 2017, American Chemical Society, Copyright 2019, Elsevier, Copyright 2019, Elsevier.

One typical example of non‐responsive hydrogels is PEG‐based injectable composite materials used for cancer therapy. In one work related to this area, Yang et al have synthesized a series of PEG‐based three‐stage copolymer composite hydrogels, referred to as “ABA”, through organic ring‐opening polymerization.^[^ ^123^ ^]^ Here, A and B refer to various units of copolymers. Such ABA copolymers can be combined with a range of biodegradable alkynes and azide‐functionalized polycarbonate. The authors investigated the efficacy of loading DOX inside the polymer chains to form micelle/hydrogel complexes through strain‐promoted alkyne‐azide cycloaddition (SPAAC). This hydrogel‐based composite shows a slow drug release rate and an improved efficiency in destroying human breast cancer cells compared with that of micellar solution formulations (**Figure** **11**).^[^ ^132^ ^]^

In a series of works carried out during the past few years, Yang et al. have reported the synthesis of a range of “abscisic acid” triblock hydrogels linked by ester blocks. However, these hydrogels typically have poor efficacy in cancer treatment, protein delivery, and immunity.^[^ ^133^, ^134^ ^]^ As such, to promote the efficacy of these triblock hydrogels, the group reported a vitamin E‐functionalized “ABA” triblock copolymer fabricated through a solvent and catalyst‐free approach based on nucleophilic addition between PEG‐diamine and vitamin E‐functionalized cyclic carbonate. Among the hydrogels developed through this strategy, the hydrogel with carbamate block connection shows promising performance in releasing the anti‐cancer antibody Herceptin, with a maximum sustained release time of up to 90 days. This type of hydrogel holds encouraging potential for inhibiting the growth of breast cancer tumors. In another work, the authors prepared a vitamin E‐PEG‐vitamin E triblock“ABA” hydrogel with greater production reproducibility and slower biodegradation.^[^ ^134^ ^]^ They introduced urethane bonds into the block connection of the “ABA” copolymer to make the hydrogel more active and stable. They used the hydrogel as a platform to deliver ovalbumin (OVA) as a model antigen for hepatitis B Vaccines. It has been shown that this hydrogel system can provide sustained antigen release and superior immune response, holding promise for the prevention of human infectious diseases such as hepatitis and human papillomavirus.

A few new preparation methods have most recently been reported for the fabrication of injectable, non‐responsive PEG hydrogels for cancer therapy. One example is the star‐shaped amphiphilic block copolymer PTMC/PEG that was synthesized by adopting a metal‐free organic catalyzed ring‐opening polymerization method, post‐polymerization chain end derivatization strategy, and self‐assembling into vesicles for the delivery of DOX in drug systems to inhibit the growth of breast cancer cells. The results showed that the loading of DOX into nano‐scale vesicle‐type hydrogels was increased by 22.5% compared with the case of micelles formed by diblock copolymer analogs. Further, DOX‐loaded vesicles show a stronger tumor growth inhibitory effect than free DOX without causing significant weight loss or cardiotoxicity.^[^ ^135^ ^]^ Another example is a cross‐linked cyanoacrylate (MOE‐CA/CA‐PEG‐CA) that was used in local sustained‐release drug delivery systems (LSRDDSs). The CA material is made of the cross‐linking agent CA‐PEG‐CA and methoxyethyl cyanoacrylate (MOE‐CA) with excellent biodegradability. MOE‐CA/CA‐PEG‐CA has been widely used for the loading of the anti‐inflammatory drug 5‐fluorouracil (J‐Fu‐1.25) to eliminate breast and gastric cancer cells.^[^ ^136^ ^]^

## 4 Commercial PEG‐Hydrogels in Cancer Therapy

Apart from academic research prospects, PEG‐hydrogels also hold remarkable commercial potential, benefiting from their simple fabrication and low cytotoxicity. As it can be understood from the research outcomes discussed in the previous sections, PEG‐hydrogels are highly attractive materials for drug delivery because of their high drug encapsulation efficiency, cumulative drug release rate, and stability in size and shape in vitro. Also, many in vivo studies have shown that the PEG hydrogel‐loaded anti‐cancer drugs can achieve the desired concentration by the rapid accumulation of PEG in the tumor tissue, thus achieving the therapeutic effect in short durations. Taken together, it can be predicted that PEG‐based hydrogels are with bright clinical application prospects in the field of cancer treatment.

PEG‐hydrogels used in anti‐tumor drug delivery with potentials in clinical applications have been developed in recent years.^[^ ^137^ ^]^ As one example, the method proposed by US Patent 6 639 014 B2 illustrates the fabrication of a biodegradable PEG‐hydrogel with highly promising application prospects for drug delivery.^[^ ^138^ ^]^ The inventors describe a PEG‐based macromer to form hydrogels that include both hydrophilic and hydrophobic fragments and cross‐linkable parts, with remarkable pH, ion concentration, and thermal responsiveness for controlled drug release. OncoGel (ReGel/paclitaxel) is an exogenous‐responsive anti‐cancer DDS composed of PLGA–PEG–PLGA triblock copolymer.^[^ ^17^ ^]^ The positive nonclinical safety and efficacy of OncoGel have been proven and it has moved on to the clinical trials. Mebiol® Gel is another example that is commercially available as a thermo‐sensitive PEG‐hydrogel product for tissue engineering and drug delivery.^[^ ^9^ ^]^

Besides, PEG‐hydrogels are also commercially promising for applications in tissue regeneration, wound healing, and antibacterial scaffolds. Trattnig et al. developed Gelrin C™ (Regentis Biomaterial Ltd.), a biosynthetic, biodegradable, PEG‐based photo‐crosslinked hydrogel, and implanted it in knee tissue with encouraging results in improving cartilage tissue repair.^[^ ^139^ ^]^ This system is currently undergoing clinical trials and is being investigated in other areas such as drug delivery. Kikgel company possesses a PEG‐hydrogel containing >90% aqua for application as a wound dressing for the treatment of ulcers, abrasions, burns, and bedsores.^[^ ^140^ ^]^ For antimicrobial applications, injectable PEG‐hydrogel product can be served for bone and wound anti‐infective dressing that controllably releases antimicrobial therapeutics, which have been demonstrated in two clinically relevant infection models.^[^ ^141^, ^142^, ^143^ ^]^

Despite all these intriguing advances in clinical applications of PEG hydrogels, the clinical studies on PEG‐hydrogel are indicative of challenges that need to be addressed in future. For instance, results from clinical trials demonstrate that the PEG nanogels can achieve a high local drug release rate; however, the complexity of body circulation overshadows its targeted accumulation percentage.^[^ ^144^ ^]^ Also, PEG has been named as the cause of an unexpected immunogenic response known as the “accelerated blood clearance (ABC) phenomenon” in clinical trials.^[^ ^145^ ^]^ The underlying reason for such immune response is that the injection of PEG can increase the level of anti‐PEG immunoglobulin, which is a soluble substance in serum.^[^ ^146^ ^]^ Suggested solutions to address this issue include manipulating the PEG particle size,^[^ ^147^ ^]^ modifying the PEG moiety (e.g., the chain length and lipid linkage),^[^ ^148^, ^149^, ^150^, ^151^, ^152^ ^]^ tuning the administration dose,^[^ ^153^ ^]^ increasing the time interval between the injections,^[^ ^154^ ^]^ and co‐delivery of immunosuppressive agents such as mitoxantrone.^[^ ^155^ ^]^

## 5 Conclusion and Future Directions

Polyethylene glycol (PEG) hydrogels are ideal candidates for developing drug delivery systems (DDSs) for cancer therapy due to their low cytotoxicity, biocompatibility, and remarkable drug‐encapsulating capability, combined with excellent circulation stability and tunability of mechanical properties. We reviewed the progress on the fabrication of PEG‐hydrogels and their application as DDSs for anti‐cancer therapy. Both non‐responsive and responsive PEG‐hydrogel DDSs were discussed, covering the systems functioning based on either exogenous stimuli‐response, such as photo‐ and magnetic‐sensitive PEG hydrogels, or endogenous stimuli‐response, such as enzyme‐, pH‐, reduction‐, and temperature‐sensitive PEG hydrogels. The review of the field highlights that both non‐responsive and stimuli‐responsive PEG‐hydrogel DDSs have shown great promise not only as DDSs but also for a wide range of other biomedical applications such as in tissue engineering, gene therapy, wound healing, and construction of antibacterial scaffolds. With continuous developments in nanotechnology, polymer material processing and characterization over the past few decades, both responsive and non‐responsive PEG‐based hydrogel nanomaterials have significantly advanced. However, their real‐world applications as DDSs are still with limitations. Stimuli‐responsive PEG‐based hydrogels have often been associated with poor response speed, sensitivity, and precision. In addition, the traditional PEG non‐responsive hydrogels show drawbacks for controlled and on‐demand release of anti‐cancer drugs. Therefore, enhancing the drug delivery performance of such hydrogels through modulating their physical and chemical structures in nano‐ and micro‐scale appears to be the central future direction in the field. A deeper knowledge of molecular chemistry combined with progression in connecting fields of biotechnology and nanomedicine presents the opportunity to innovate through the design and creation of PEG‐based hydrogels to produce more potent drug delivery systems for cancer therapy and beyond.

## Conflict of Interest

The authors declare no conflict of interest.
